# Cytokine and Haptoglobin Profiles From Shipping Through Sickness and Recovery in Metaphylaxis- or Un-Treated Cattle

**DOI:** 10.3389/fvets.2021.611927

**Published:** 2021-03-19

**Authors:** Carol G. Chitko-McKown, Gary L. Bennett, Larry A. Kuehn, Keith D. DeDonder, Michael D. Apley, Gregory P. Harhay, Michael L. Clawson, Aspen M. Workman, Bradley J. White, Robert L. Larson, Sarah F. Capik, Brian V. Lubbers

**Affiliations:** ^1^USDA-ARS, US Meat Animal Research Center, Clay Center, NE, United States; ^2^College of Veterinary Medicine, Kansas State University, Manhattan, KS, United States

**Keywords:** cytokines, interleukin-1β, interleukin-6, tumor necrosis factor-α, cattle, haptoglobin, bovine respiratory disease complex, metaphylaxis

## Abstract

Fifty-six head of cattle, 28 animals with bovine respiratory disease complex (BRDC), and 28 healthy animals that were matched by treatment, sale barn of origin, day, and interactions among these variables, were identified from a population of 180 animals (60 each purchased at three sale barns located in Missouri, Tennessee, and Kentucky) enrolled in a study comparing animals receiving metaphylaxis to saline-treated controls. Cattle were transported to a feedlot in KS and assigned to treatment group. Blood samples were collected at Day 0 (at sale barn), Day 1, Day 9, and Day 28 (at KS feedlot), and transported to the US Meat Animal Research Center in Clay Center, NE where plasma was harvested and stored at −80°C until assayed for the cytokines IFN-γ, IL-1β, IL-6, and TNF-α, and the acute stress protein haptoglobin (HPT). Our objectives were to determine if cytokine and haptoglobin profiles differed between control and metaphylaxis treatment groups over time, and if profiles differed between animals presenting with BRDC and those that remained healthy. There was no difference between the treated animals and their non-treated counterparts for any of the analytes measured. Sale barn of origin tended to affect TNF-α concentration. Differences for all analytes changed over days, and on specific days was associated with state of origin and treatment. The Treatment by Day by Case interaction was significant for HPT. The analyte most associated with BRDC was HPT on D9, possibly indicating that many of the cattle were not exposed to respiratory pathogens prior to entering the feedlot.

## Introduction

Bovine respiratory disease complex (BRDC) remains a serious health and economic problem for cattle producers despite the availability of vaccines and widespread use of antibiotics. This disease complex is the result of interactions between the host, viruses, bacteria, and the environment. It is frequently characterized by a primary viral infection in combination with stress that suppresses the host's immune system allowing opportunistic bacteria to infect the lung. Changes in innate defenses, bacterial colonization, leukocyte recruitment to the lung, and the induction of pro-inflammatory cytokines such as TNF-α and IL-6 culminate in the pathology associated with BRDC (1). Predisposing factors that increase the likelihood of an animal developing respiratory disease include weaning, shipping, weather, source, and viral infections (2). Stress of any kind may affect respiratory immunity (3), thus predisposing an animal to BRDC. Greater variability in serum inflammatory cytokine concentrations exist among calves in response to weaning even prior to additional transportation or treatment (4). A reduction of the prevalence, severity and/or treatment of respiratory diseases would enhance producer efficiency and promote the welfare of livestock.

Bovine bronchial epithelial cells simultaneously exposed to the respiratory pathogens bovine herpesvirus−1 and *Mannheimia haemolytica* express the inflammatory cytokines IL-1β and TNF-α however, cells exposed to *M. haemolytica* alone additionally expressed IFN-γ (5). When bovine alveolar macrophages are stimulated with lipopolysaccharide from *M. haemolytica*, TNF-α and IL1-β mRNA is detectable within 30 min, and secreted protein for TNF-α peaked at 4 h and for IL1-β peaked at 8 h indicating that these inflammatory cytokines may play a role in the pathogenesis of lung injury in BRDC (6). Similarly, calves experimentally infected with bovine respiratory syncytial virus (BRSV) produced TNF-α in their lungs (7). Acute phase proteins such as haptoglobin (HPT) are produced in the liver in response to inflammatory cytokine such as IL-1β and TNF-α and have been used to differentiate between chronic and acute inflammation in cattle (8, 9). Several groups have found HPT concentrations to be a useful biomarker for inflammation due to BRDC (10–12). Grell et al. reported an increase in the expression of IL-6, HPT, and IFN-γ following infection with BRSV (13), and Heegard also found increases in HPT in infected animals, that remained low in all control animals (14). Wernicki et al. measured HPT as an indicator of shipping stress resulting in respiratory disease in feedlot calves (15).

Information on the association of on-farm arrival data and health and performance of calves is limited (9). By measuring levels of cytokines associated with inflammation or a response to viral pathogens combined with acute phase proteins, it may be possible to determine a profile of circulating analytes that might indicate a calf may be at risk of presenting with BRDC prior to showing characteristic clinical signs of lethargy and fever. Our objective was to sample calves from day of purchase at one of three sale barns through 28 days at a feedlot to determine if changes in the concentrations of cytokines and HPT measured over time would be different in animals presenting with BRDC and those that remained healthy. This would possibly result in a panel of biomarkers that could be used in the future to identify animals at risk for BRDC prior to or upon arrival at a feedlot so that specific measures could be taken to prevent BRDC or to improve their health and productivity.

## Materials and Methods

All activities were reviewed and approved by the Kansas State University Institutional Animal Care and Use Committee (#3338).

### Animals

Cattle enrolled in the study were part of a randomized clinical trial to evaluate the metaphylactic and therapeutic effects as well as the pharmacokinetics and pharmacodynamics of gamithromycin in naturally occurring bovine respiratory disease (16, 17). One hundred eighty head of cattle at high risk for developing BRDC were purchased at sale barns in three states-−60 castrated, male, mixed-breed cattle each from Richmond, Kentucky; Maryville, Missouri, and Athens, Tennessee—and were shipped to a feedlot in Manhattan, Kansas, at distances of 726, 172, and 841 miles respectively. Cattle ranged in weight from 362 to 592 pounds with an average weight of 470 pounds. Upon arrival at the feedlot the animals were processed and separated into two pens per state based on treatment. One group received metaphylaxis with gamithromycin (MET) and one group received saline (CO). All animals received a modified live viral respiratory vaccine, a clostridial vaccine, anthelminthic, growth implant and were examined to ensure that no clinical signs of BRDC were present upon intake. Animals were then observed daily by a veterinarian masked to treatment assignment for signs of BRDC (16–18). No animals died during the course of this study.

### Blood Sampling

Whole blood was collected into 10 ml Vacutainers containing ethylenediaminetetraacetic acid as an anticoagulant (BD, Franklin Lakes, NJ; #366643) via jugular venipuncture from cattle at the point of purchase at each sale barn (D0), upon arrival and processing at the feedlot (D1), eight days after arrival at the feedlot (D9) and 27 days after arrival (D28). Blood was kept on wet ice and was immediately transported to the US Meat Animal Research Center in Clay Center, NE for arrival within 24H. The blood samples were then immediately logged, followed by centrifugation at 1,200 X g for 20 min to separate plasma. Plasma was removed and aliquoted into 2 or 3 cryovials, depending upon actual sample volume, for storage at −80°C. Each of the three sets of plasma samples were stored in different ultracold freezers to protect against sample degradation in the event of an individual freezer's failure.

### Animal Matching

For our study, cattle that were diagnosed with BRDC were retrospectively matched at the end of the study with cohorts from the same sale barn of origin (SBO), pen, location in transport trailer, and treatment (MET or CO) that showed no signs of disease for the entire length of the study. The purpose of matching was to reduce the number of assays run while balancing origin, pen, and treatment effects of sick and healthy animals. These animals were used this study's population of 28 BRDC-infected and 28 healthy cattle.

### Metabolite Analyses

The cytokines IFN-γ, IL-1β, IL-6, and TNF-α were analyzed using Meso Scale Diagnostics (MSD, Rockville, MD) assays in duplicate. Bovine IFN-γ was analyzed using a traditional MSD platform with plates custom printed by the company with a proprietary anti-bovine IFN-γ antibody. Briefly, plates were blocked, washed, and the standard (recombinant bovine IFN-γ; 5000–0 pg/ml) and bovine plasma samples were added and incubated for 2 h. Plates were washed, and a biotinylated secondary antibody bound to Sulfo-Tag streptavidin (MSD) was added and incubated for 2 h. Plates were washed a final time and MSD Read Buffer T (MSD) was added and the plate read on a MESO QuickPlex SQ120 imager and data analyzed using MSD WorkBench 4.0 software (MSD).

IL-1β, IL-6, and TNF-α were analyzed concurrently using the MSD U-PEX platform (#K15228N-2). This multiplex assay was developed in our lab using “Do-It-Yourself” ELISA kits purchased from Kingfisher Biotech, Inc. (St. Paul, MN; DIY0675B-003, DIY0670B-003, DIY1111B-003). The assays were run per the manufacturer's instructions essentially as described above. However, after blocking, the biotinylated polyclonal antibody from the kit was coupled to a linker specific for one of 10 locations within each well of the plate. The plate was then treated as described above. Standards/calibrators were prepared by 4-fold serial dilutions of the recombinant cytokines in dilution buffer, with the final standard being diluent alone. Starting concentrations for the three recombinant bovine cytokines were as follows: IL-1β, 50,000 pg/ml, IL-6, 10,000 pg/ml, and TNF-α, 50,000 pg/ml. Samples falling outside of the linear range of the standard curve were diluted and reanalyzed.

Bovine Haptoglobin (HPT) was analyzed using a traditional sandwich assay (Immunology Consultants Laboratory, Inc., Portland, OR, #E-10HPT), as per the manufacturer's instructions. Standards and plasma samples were applied to a 96-well plate coated with anti-bovine HPT antibody. Standards were run from 1,000 ng/ml to 0 ng/ml. After a 15 min incubation at room temperature, plates were washed and enzyme-conjugated antibody to bovine HPT was added and the plate incubated for 15 min at room temperature. Plates were washed and enzyme substrate was added for 10 min followed by stop solution. Absorbance was determined by reading the plate at 450 nm on a traditional microplate reader. Concentrations were determined by four parameter logistic curve fitting (http://www.ELISAanalysis.com). Samples falling outside of the linear range of the standard curve were diluted and reanalyzed.

### Statistical Analysis

Natural logarithms of concentrations of cytokines and haptoglobin for each time point were analyzed with PROC GLIMMIX in SAS version 9.4 (SAS Inc., Cary, NC). Fixed effects for treatment (MET, or CO), SBO (Kentucky, Missouri, or Tennessee), day on experiment (0, 1, 9, 28), and case (presenting with BRDC sometime during the 28 days or healthy) were fitted to test average differences in these effects. Consistency of differences across day were tested by fitting interactions of day with case, treatment, SBO, and Treatment X Case. Random effects in the model were experimental pen, assay plate, animal, and residual. Animal effects measure consistency of concentrations across days. Repeatability of animal concentrations across days was estimated from animal variance divided by the sum of animal and residual variance. Repeatability was considered significant when animal variance was significant. Results were considered highly significant with *P* = 0.01, significant with *P* = 0.05, and trending with *P* = 1.0.

## Results and Discussion

### Animals Presenting With BRDC and Their Matched Counterparts

Of the 180 head of cattle enrolled in the parent study purchased from sale barns in three different states (Kentucky, Missouri, or Tennessee) and enrolled in two treatments (MET or CO), 28 animals presented with BRDC within the 28 days of the study (18). Of these 28 animals, nine were in the MET treatment group (1 from Kentucky, 1 from Missouri, and 7 from Tennessee) and 19 were in the CO treatment group (9 from Kentucky, 8 from Missouri, and 2 from Tennessee). These animals were matched to healthy cohorts to determine if differences in cytokine and HPT concentrations over time could be identified between cattle with BRDC and healthy pen-mates. The small number of cattle with BRDC and to a lesser extent the imbalance between numbers of MET and CO animals limits the ability to find statistically significant differences. Additionally, we analyzed cytokine and HPT profiles between animals in the MET treatment with animals in the CO treatment group. Repeatabilities of cytokine concentrations were significant and moderate for IFN-γ (0.25), IL-1β (0.53), IL-6 (0.44), and TNF-α (0.53), but not for HPT concentration (0.07). These repeatabilities are consistent with HPT responses being temporal and transitory and cytokine concentrations being partially due to innate or other longer lasting factors.

**Figure 1:**
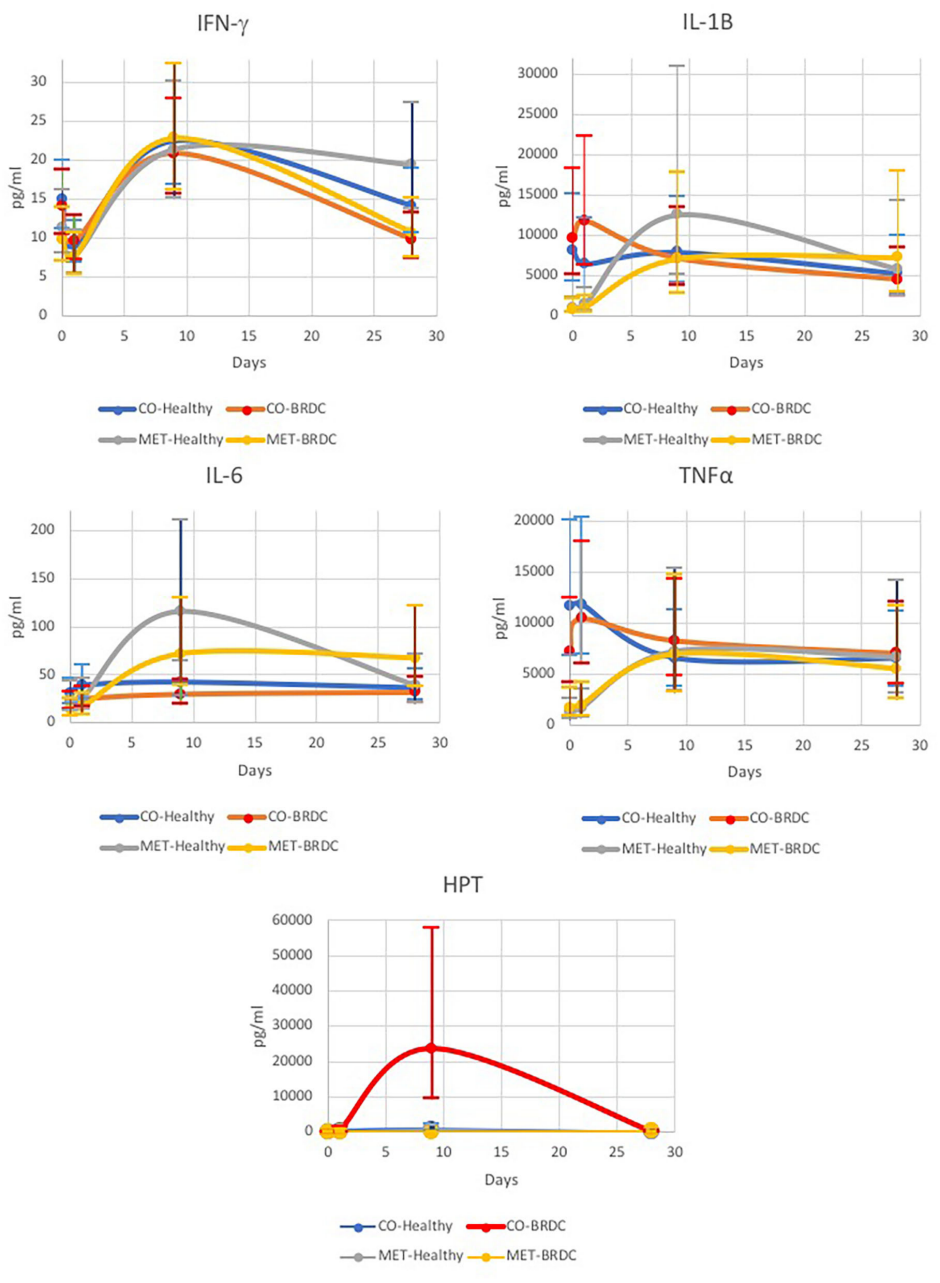
Concentrations of bovine IFN-γ, IL-1β, IL-6, TNF-α, and haptoglobin (HPT) measured over time for cattle diagnosed with BRDC (*n* = 28) and healthy cattle (*n* = 28).

IFN-γ was significantly different over the days of the experiment, and was highest on D9 (P < 0.010), however, both healthy and animals that presented with BRDC had higher concentrations at this time (Table 1 and Figure 1). In the parent study the mean and median dates of BRDC diagnosis were equal at 14 days (17). When the Case X Day interaction was examined, IFN-γ was higher on D28 for healthy animals than for animals presenting with BRDC (*P* = 0.08; Table 1). This may indicate a more robust immune response in those animals that did not become ill.

**Table 1:** Bovine cytokine and haptoglobin concentrations.

**Analyte**			**IL-1β**			**TNF-α**			**IL-6**			**IFN-γ**			**Haptoblobin**		
			**pg/ml**	**ln value ± SE**	**P value**	**pg/ml**	**ln value ± SE**	**P value**	**pg/ml**	**ln value ± SE**	**P value**	**pg/ml**	**ln value ± SE**	**P value**	**ng/ml**	**ln value ± SE**	**P value**
**Treatment**					0.20			0.12			0.74			0.85			0.19
CO			7266	8.89 ± 0.46		8444	9.04 ± 0.41		31.5	3.44 ± 0.30		13.66	2.61 ± 0.26		374	5.92 ± 0.51	
MET			2798	7.93 ± 0.63		3226	8.07 ± 0.55		36.6	3.59 ± 0.40		12.68	2.53 ± 0.28		116	4.75 ± 0.68	
**Sale barn of origin**					0.24			0.06			0.82			0.73			0.61
Kentucky			2268	7.72 ± 0.62		2564	7.84 ± 0.54		28.6	3.35 ± 0.40		14.07	2.64 ± 0.34		330	5.79 ± 0.72	
Missouri			4439	8.39 ± 0.63		4280	8.36 ± 0.55		36.2	3.58 ± 0.40		10.67	2.36 ± 0.34		126	4.83 ± 0.73	
Tennessee			9107	9.11 ± 0.57		12953	9.46 ± 0.50		37.8	3.63 ± 0.36		15.18	2.71 ± 0.32		218	5.38 ± 0.67	
**Day**					<0.01			0.03			<0.01			<0.01			<0.01
0			2747	7.91 ± 0.46		3613	8.19 ± 0.41		21.4	3.06 ± 0.31		12.39	2.51 ± 0.21		36	3.57 ± 0.58	
1			3198	8.07 ± 0.46		4462	8.40 ± 0.41		25.7	3.24 ± 0.31		8.46	2.13 ± 0.21		330	5.79 ± 0.58	
9			8389	9.03 ± 0.46		7203	8.88 ± 0.41		57.2	4.04 ± 0.31		21.92	3.08 ± 0.21		867	6.76 ± 0.58	
28			5611	8.63 ± 0.46		6389	8.76 ± 0.41		42.1	3.73 ± 0.31		13.06	2.56 ± 0.21		184	5.21 ± 0.58	
**Case**					0.95			1.00			0.37			0.22			0.34
Healthy			4583	8.43 ± 0.49		5219	8.56 ± 0.43		39.1	3.66 ± 0.31		14.14	2.64 ± 0.21		163	5.09 ± 0.48	
BRDC			4437	8.39 ± 0.49		5219	8.56 ± 0.43		29.5	3.3 ± 0.31		12.25	2.50 ± 0.20		266	5.58 ± 0.48	
**Sale barn of origin** **×** **Day**	**Day**				0.12			0.07			<0.01			0.69			0.12
Kentucky	0		1009	6.91 ± 0.72		1300	7.16 ± 0.62		12.8	2.55.± 0.47		13.94	2.63 ± 0.36		27	3.31 ± 1.05	
Missouri	0		2178	7.68 ± 0.73		2399	7.78 ± 0.62		28.2	3.33 ± 0.48		9.77	2.27 ± 0.36		39	3.66 ± 1.06	
Tennessee	0		9431	9.15 ± 0.66		15131	9.62 ± 0.57		27.2	3.30 ± 0.44		13.96	2.63 ± 0.34		43	3.75 ± 0.97	
Kentucky	1		1329	7.19 ± 0.72		2000	7.60 ± 0.61		18.0	2.89 ± 0.47		10.11	2.31 ± 0.36		403	5.99 ± 1.04	
Missouri	1		2036	7.61 ± 0.74		2480	7.81 ± 0.63		26.7	3.28 ± 0.48		7.21	1.97 ± 0.36		56	4.03 ± 1.06	
Tennessee	1		12088	9.4 ± 0.66		17908	9.79 ± 0.57		35.2	3.56 ± 0.44		8.30	2.11 ± 0.34		1577	7.36 ± 0.97	
Kentucky	9		5381	8.59 ± 0.72		5200	8.55 ± 0.61		38.9	3.66 ± 0.47		20.26	3.00 ± 0.36		1177	7.07 ± 1.04	
Missouri	9		8949	9.09 ± 0.73		6752	8.81 ± 0.62		92.4	4.5 ± 0.48		17.30	2.85 ± 0.36		689	6.53 ± 1.06	
Tennessee	9		12261	9.41 ± 0.67		10642	9.27 ± 0.57		52.1	3.95 ± 0.44		30.05	3.40 ± 0.34		804	6.68 ± 0.97	
Kentucky	28		3668	8.20 ± 0.72		3198	8.07 ± 0.61		73.8	4.30 ± 0.47		13.74	2.62 ± 0.36		909	6.81 ± 1.04	
Missouri	28		9784	9.18 ± 0.73		8354	9.03 ± 0.62		24.6	3.20 ± 0.48		10.64	2.36 ± 0.36		164	5.10 ± 1.06	
Tennessee	28		4921	8.50 ± 0.66		9762	9.18 ± 0.57		41.1	3.71 ± 0.44		15.24	2.72 ± 0.34		42	3.73 ± 0.98	
**Day** **×** **Case**	**Case**				0.89			0.99			0.37			0.08			0.98
0	Healthy		2668	7.88 ± 0.56		3733	8.22 ± 0.48		27.1	3.29 ± 0.37		13.06	2.56 ± 0.22		25	3.22 ± 0.74	
0	BRDC		2828	7.94 ± 0.56		3497	8.15 ± 0.49		16.9	2.82 ± 0.37		11.76	2.46 ± 0.22		51	3.9 ± 0.75	
1	Healthy		3015	8.01 ± 0.56		4413	8.39 ± 0.48		32.0	3.46 ± 0.37		8.40	2.12 ± 0.22		286	5.65 ± 0.74	
1	BRDC		3391	8.12 ± 0.56		4512	8.41 ± 0.49		20.6	3.02 ± 0.37		8.51	2.14 ± 0.22		380	5.94 ± 0.74	
9	Healthy		9932	9.20 ± 0.56		6860	8.83 ± 0.48		70.5	4.25 ± 0.37		21.96	3.08 ± 0.22		735	6.59 ± 0.74	
9	BRDC		7086	8.86 ± 0.56		7563	8.93 ± 0.48		46.5	3.83 ± 0.37		21.88	3.08 ± 0.22		1023	6.93 ± 0.74	
28	Healthy		5520	8.61 ± 0.56		6567	8.78 ± 0.48		38.2	3.64 ± 0.37		16.59	2.80 ± 0.22		134	4.89 ± 0.74	
28	BRDC		5703	8.64 ± 0.56		6217	8.73 ± 0.48		46.4	3.83 ± 0.37		10.28	2.33 ± 0.22		254	5.53 ± 0.76	
**Treatment** **×** **Day**	**Day**				<0.01			<0.01			0.07			0.29			0.12
CO	0		8805	9.08 ± 0.52		9166	9.12 ± 0.46		25.4	3.23 ± 0.35		14.50	2.67 ± 0.27		45	3.79 ± 0.72	
CO	1		8705	9.07 ± 0.52		11138	9.31 ± 0.46		31.6	3.45 ± 0.35		9.37	2.23 ± 0.27		657	6.48 ± 0.72	
CO	9		7480	8.92 ± 0.52		7358	8.90 ± 0.46		35.8	3.57 ± 0.35		21.69	3.07 ± 0.27		4822	8.48 ± 0.72	
CO	28		4861	8.48 ± 0.52		6767	8.81 ± 0.46		34.3	3.53 ± 0.35		11.82	2.46 ± 0.27		138	4.92 ± 0.72	
MET	0		857	6.75 ± 0.74		1424	7.26 ± 0.63		18.1	2.89 ± 0.49		10.59	2.35± 0.31		29	3.35 ± 1.05	
MET	1		1175	7.06 ± 0.74		1787	7.48 ± 0.63		20.9	3.04 ± 0.49		7.63	2.03 ± 0.31		165	5.10 ± 1.04	
MET	9		9409	9.14 ± 0.74		7050	8.86 ± 0.63		91.5	4.51 ± 0.49		22.15	3.09 ± 0.31		156	5.04 ± 1.04	
MET	28		6476	8.77 ± 0.74		6032	8.70 ± 0.63		51.6	3.94 ± 0.49		14.44	2.66 ± 0.31		247	5.50 ± 1.05	
**Treatment** **×** **Day** **×** **Case**	**Day**	**Case**			0.85			0.79			0.87			0.93			0.05
CO	0	Healthy	8065	8.99 ± 0.63		11663	9.36± 0.54		30.8	3.42 ± 0.42		14.96	2.70 ± 0.29		29	3.38 ± 0.89	
CO	0	BRDC	9613	9.17 ± 0.64		7203	8.88 ± 0.55		20.9	3.04 ± 0.42		14.05	2.64 ± 0.29		68	4.21 ± 0.91	
CO	1	Healthy	6427	8.76 ± 0.63		11877	9.38 ± 0.54		39.4	3.67 ± 0.42		9.10	2.20 ± 0.29		671	6.50 ± 0.89	
CO	1	BRDC	11790	9.37 ± 0.64		10444	9.25 ± 0.55		25.3	3.23 ± 0.42		9.65	2.26 ± 0.29		643	6.46 ± 0.89	
CO	9	Healthy	7863	8.96 ± 0.63		6524	8.78 ± 0.54		42.7	3.75 ± 0.42		22.54	3.11 ± 0.29		977	6.88 ± 0.89	
CO	9	BRDC	7117	8.87 ± 0.63		8299	9.02 ± 0.54		30.0	3.40 ± 0.42		20.88	3.03 ± 0.29		23811	10.07 ± 0.89	
CO	28	Healthy	5251	8.56 ± 0.63		6494	8.77 ± 0.54		36.9	3.60 ± 0.42		14.19	2.65 ± 0.29		96	4.56 ± 0.89	
CO	28	BRDC	4500	8.41 ± 0.63		7052	8.86 ± 0.54		31.9	3.46 ± 0.42		9.84	2.28 ± 0.29		197	5.28 ± 0.89	
MET	0	Healthy	883	6.78 ± 0.91		1195	7.08 ± 0.76		23.9	3.17 ± 0.59		11.40	2.43 ± 0.35		22	3.07 ± 1.30	
MET	0	BRDC	832	6.72 ± 0.91		1698	7.43 ± 0.76		13.7	2.6 ± 0.59		9.83	2.28 ± 0.35		38	3.63 ± 1.30	
MET	1	Healthy	1415	7.25 ± 0.91		1639	7.40 ± 0.76		26.0	3.25 ± 0.59		7.76	2.04 ± 0.35		122	4.80 ± 1.30	
MET	1	BRDC	975	6.88 ± 0.91		1949	7.5 ± 0.76		16.8	2.82 ± 0.59		7.51	2.01 ± 0.35		225	5.41 ± 1.30	
MET	9	Healthy	12547	9.43 ± 0.91		7212	8.88 ± 0.76		116.5	4.75 ± 0.59		21.40	3.06 ± 0.35		553	6.31 ± 1.30	
MET	9	BRDC	7056	8.86 ± 0.92		6892	8.83 ± 0.76		71.9	4.27 ± 0.59		22.92	3.13 ± 0.35		44	3.78 ± 1.30	
MET	28	Healthy	5803	8.66 ± 0.91		6641	8.8 ± 0.76		39.5	3.67 ± 0.59		19.41	2.96 ± 0.35		187	5.22 ± 1.30	
MET	28	BRDC	7228	8.88 ± 0.91		5480	8.60 ± 0.76		67.6	4.21 ± 0.59		10.74	2.37 ± 0.35		326	5.78 ± 1.34	

IL-1β was significantly different for Day (*P* < 0.01) with the highest concentration of IL-1β occurring on D9 (Table 1 and Figure 1), and similar to IFN-γ, both healthy animals and those that presented with BRDC had higher concentrations at this time. Levels remained high on D28 possibly due to continued exposure to pathogens in the feedlot environment. The Treatment X Day interaction was also highly significant (*P* < 0.01) for IL-1β (Table 1). Control animals were highest at D0 and D1, and the concentration decreased over time. In MET animals, the reverse was noted with D0 having the lowest concentrations of IL-1β followed by D1. The highest concentrations of this cytokine were measured on D9 in the MET animals, possibly due to immunomodulatory properties of macrolides (Table 1 and Figure 1).

Differences in IL-6 concentrations were significant for Day (*P* < 0.01), and for the Day X SBO interaction (*P* < 0.01), and showed a trend in the Treatment X Day interaction (*P* = 0.07) (Table 1). IL-6 concentrations peaked on D9, similar to IFN-γ and IL-1β, and remained higher at D28 than on D0 and D1 (Figure 1). IL-6 concentration was lowest in animals from the Kentucky sale barn on D0, D1, and D9, but had the highest concentration on D28. Animals from the Missouri sale barn had the highest IL-6 concentrations on D0 and D9, but the lowest on D28, and animals from the Tennessee sale barn had the highest concentrations of IL-6 on D1, but the median IL-6 concentration for all other days. For Treatment X Day, the lowest IL-6 concentration was noted in MET animals on D0, however these same animals had the highest IL-6 concentration on D9 (Figure 1).

Concentrations of TNF-α were significantly different on Day (*P* = 0.03), and Treatment X Day (*P* < 0.01; Table 1). TNF-α was lowest on D0 and increased to D9 and then decreased by D28 (Figure 1). When broken down by CO vs. MET, the CO animals had the highest TNF-α concentrations on D0 and D1 in contrast to the MET group that had the lowest concentrations on these 2 days prior to treatment with gamithromycin (Figure 1). The TNF-α concentrations in the MET animals peaked at D9 then decreased somewhat at D28, however, this level was higher than on those measured on D0 and D1. Sale barn of origin showed a tendency (*P* = 0.07) for animals purchased in Kentucky to have the lowest TNF-α concentrations and those purchased in Tennessee to have the highest. This pattern was observed on all four sampling days (Table 1).

HPT concentration differences were highly significant by Day (*P* = 0.01) with concentrations being lowest at D0 and peaking at D9 (Table 1). The interaction of Treatment X Day X Case was also significant (P = 0.05) with concentrations being highest for CO animals with BRDC on D9 (Figure 1). This may be due to the extremely high concentrations of HPT measured for animals in the CO treatment that later presented with BRDC. Wernicki et al. also found the highest HPT concentrations in feedlot calves at D9 after arrival in both control and stressed animals, however the stressed animals had concentrations more than three-fold greater than the control animals (15). Interestingly, the BRDC-affected MET animals on D9 had lower HPT concentrations than their healthy counterparts (Figure 1). This is similar to results found by Celestino et al. in a study comparing metaphylactic strategies to control treatment in week old dairy calves (19). One subcutaneous injection of tildipirosin at enrollment followed by a subsequent injection 17 days later resulted in a minimal change in HPT concentration in the calves at 27 days post enrollment (19), possibly indicating an anti-inflammatory effect of metaphylaxis (16). Moisa et al. found that HPT values were higher in calves with BRDC however, there was greater variability in the HPT concentration in plasma from these calves than from the control animals, and that concentration also varied by location (12).

Environmental conditions and the immunological status of an animal undoubtedly play a role in disease outcome (8, 16). Additionally, different metaphylactic treatments and strategies result in different levels of mortality and morbidity in stocker and feedlot cattle (20), however, these treatments are generally found to reduce morbidity (2). Some approaches to the early identification of cattle that will soon show signs of BRDC include the use of infrared thermography (21), modeling based on feedlot arrival data (22), and the use of biomarkers to predict disease outcome (23). We did not attempt to identify profiles associated with mortality because all animals in our study were successfully treated, and no animals died while on study. Roe has suggested an inflammation classification system using cytokine parameters (24). The cytokines included for bacterial inflammation include IL-1β, TNF-α, IL-6, and IL-8; and for viral inflammation these along with IL-12, IFN-γ and IFN-α. Our panel included four out of these seven cytokines and also included HPT. Thus far no single indicator of disease has been identified (2).

In conclusion, our results indicate that the analyte most closely associated with subsequent diagnosis with BRDC was HPT on D9, possibly indicating that a high proportion of the cattle were not exposed to respiratory pathogens prior to entering the feedlot environment. This is supported by the low percentage of morbidity in the majority of animals in the study population throughout the 28 day study period. However, a measurement obtained on D9 would not assist in selecting treatment prior to arriving at the feedlot, and additional handling on D9 might prove to be more stressful, less efficient for the feedlot operators, and possibly counterproductive.

## Data Availability Statement

The raw data supporting the conclusions of this article will be made available by the authors, without undue reservation.

## Ethics Statement

The animal study was reviewed and approved by Kansas State University Institutional Animal Care and Use Committee.

## Author Contributions

The animal study was designed by KD, MA, GH, MC, BW, RL, SC, and BL. Sample collection was provided by KD, MA, BW, RL, SC, and BL. Sample analysis was provided by CC-M. Data analysis was provided by GB, LK, and AW. CC-M drafted the manuscript with the assistance and review by all authors.

## Conflict of Interest

KD is currently employed at the Veterinary & Biomedical Research Center, Inc., Manhattan, KS, United States. This commercial affiliation began after the research presented in this manuscript was completed. The remaining authors declare that the research was conducted in the absence of any commercial or financial relationships that could be construed as a potential conflict of interest.
